# Phase II study of induction chemotherapy followed by chemoradiotherapy in patients with borderline resectable and unresectable locally advanced pancreatic cancer

**DOI:** 10.1038/srep45845

**Published:** 2017-04-05

**Authors:** Michele Fiore, Sara Ramella, Sergio Valeri, Damiano Caputo, Barnaba Floreno, Pasquale Trecca, Luca Eolo Trodella, Lucio Trodella, Rolando Maria D’Angelillo, Roberto Coppola

**Affiliations:** 1 Radiotherapy Unit, Campus Bio-Medico University, Rome, Italy; 2 Department of General Surgery, Campus Bio-Medico University, Rome, Italy

## Abstract

There is not a clear consensus regarding the optimal treatment of locally advanced pancreatic disease. There is a potential role for neoadjuvant therapy to treat micrometastatic disease with chemotherapy, as well as for the treatment of local disease with radiotherapy. We evaluated the safety and efficacy of induction chemotherapy with oxaliplatin and gemcitabine followed by a high weekly dose of gemcitabine concurrent to radiation therapy in patients with borderline resectable and unresectable locally advanced pancreatic cancer. In our study, 41 patients with pancreatic cancer were evaluated. In all cases an accurate pre-treatment staging was performed. Patients with evidence of metastatic disease were excluded, and thus a total of 34 patients were consequently enrolled. Of these, twenty-seven patients (80%) had locally advanced unresectable tumours, seven patients (20%) had borderline resectable disease. This protocol treatment represents a well-tolerated promising approach. Fifteen patients (55.5%) underwent surgical radical resection. With a median follow-up of 20 months, the median PFS and OS were 20 months and 19.2 months, respectively. The median OS for borderline resectable patients was 21.5 months compared with 14 months for unresectable patients (p = 0.3). Continued optimization in multimodality therapy and an accurate patient selection remain crucial points for the appropriate treatment of these patients.

Pancreatic cancer is the fourth leading cause of cancer death in the U.S. An estimated 53,070 new cases were diagnosed and 41,780 deaths were expected in the U.S. in 2016^1^. Surgery is the only potentially curative treatment, but it is confined to 20% of patients with clinically localized disease. Unfortunately, the majority of patients present with borderline resectable or unresectable pancreatic cancer^2^. Despite advances in radiation therapy techniques and improved chemotherapeutic regimes, the poor prognosis of patients with pancreatic cancer has not significantly improved. There is no a clear consensus regarding the optimal treatment of locally advanced pancreatic disease. The two most commonly selected therapeutic options are gemcitabine-based chemotherapy and radiochemotherapy (RCT)^3^. During the last decade, several studies have tested the combination of both approaches as a sequential schedule of induction chemotherapy followed by RCT for patients without evidence of progression^4^,^5^,^6^,^7^. This multidisciplinary treatment strategy allows not only the treatment of systemic disease upfront, but also provides the opportunity for early detection of a rapidly aggressive disease that would not benefit from RCT. In order to better select patients for treatment and to improve clinical outcomes, we designed a prospective phase II study to evaluate the tolerability and efficacy of induction chemotherapy with oxaliplatin and gemcitabine followed by a high weekly dose of gemcitabine concurrent to radiation therapy in borderline resectable (BRCP) and unresectable locally advanced pancreatic cancer (LAPC).

## Materials and Methods

This trial was performed as a single-center one-armed phase II study. Forty-one treatment-naïve patients affected by histologically proven pancreatic cancer, with borderline resectable or locally advanced disease, were included in the study. Definitions of borderline and unresectable disease were as per NCCN guidelines. Borderline resectable tumors were defined by venous involvement of the superior mesenteric vein (SMV) or portal vein (PV), gastroduodenal artery encasement, or abutment of the superior mesenteric artery (SMA) up to 180°. Unresectable disease was defined by greater than 180° of SMA involvement, SMV/PV occlusion that is not amenable to reconstruction, or aortic or inferior vena cava (IVC) invasion or encasement. The selected patients did not have a history of metastatic cancer, prior history of radiotherapy to the abdomen, or younger than 18 years of age. Only patients with Eastern Cooperative Oncology Group (ECOG) performance status 0 or 1 and hematologic parameters that indicated adequate bone marrow, renal and hepatic function were considered eligible. In all cases a thorough pre-treatment staging was performed, including: physical examination, complete blood tests and tumor markers, endoscopic ultrasonography (EUS) with fine needle aspiration biopsy, multilayer CT scan, PET-CT with 18F-2-fluoro-2-deoxy-D-glucose (FDG) and laparoscopy with peritoneal washing. Jaundiced patients before or during treatment underwent endoscopic biliary stenting. All patients signed a study specific consent form.

The induction phase of the treatment plan was designed to administer gemcitabine 1000 mg/mq and oxaliplatin 100 mg/mq every 14 days for four doses. For patients without disease progression as detected through restaging exams (CT scan, 18-FDG PET-CT scan), chemotherapy was followed by RCT which consisted of conformal radiation therapy and concurrent gemcitabine at the dose of 600 mg/mq weekly. In the combined phase of the treatment all patients underwent simulation by using a Siemens 16-CT simulator (Siemens Medical System). Radiotherapy target volumes were established by CT scan and PET-CT scan. For patients with BRPC, the Clinical Target Volume (CTV) included the tumour and involved lymph nodes (>1 cm on CT scan and PET positive); the external beam radiation was delivered with a total dose of 54 Gy with fractionation of 1.8 Gy daily for 5 days a week. For patients with LAPC, the CTV included tumour and involved lymph nodes for a total dose of 59.4 Gy, targeting also frequently involved peripancreatic lymph nodes at risk for the dose of 45 Gy, with fractionation of 1.8 Gy daily for five days a week. In both cases, the Planning Target Volume (PTV) was defined by CTV with a safety margin of 1 cm in all directions to include organ motion and set-up errors. Patients were fixed during therapy by individual immobilization devices. Patients were treated using 3D-conformal radiotherapy. Organs at risk for radiation-induced side effects were contoured on the dose planning CT and dose volume histograms (DVH) were calculated. Doses to the liver, kidneys, stomach and spine did not exceed the tolerance of these normal tissues. All treatments were delivered with a 15-MV linear accelerator (Varian Medical System) with a multifield isocentric technique using a multileaf collimator. All fields were treated daily. A quality-control protocol was applied for all patients with periodical digital portal images to evaluate the precision of the set-up. During treatment, patients were evaluated through a directed history and weekly physical examination. The occurrence and nature of any adverse events were recorded in accordance with the National Cancer Institute Common Toxicity Criteria (version 4.0) scale.

When multiple treatment-related adverse events of the same type occurred in the same patient, only the most severe ones were reported. Subsequently, the dose of chemotherapy was adjusted according to the number of occurrences of grade 2 or greater events: after a first occurrence, 75% of starting dose was used, while after a second occurrence, 50% of the starting dose was used.

Four weeks after the completion of RCT, restaging, consisting of clinical examination, laboratory test, tumor markers, CT scan and PET-CT scan, was performed. Tumor response was defined in accordance with the World Health Organization (WHO) definition through CT scan and PET-CT scan. Surgery was considered in patients whose tumors were technically resectable.

After resection, patients were evaluated every three months by means of a standard surveillance protocol that included history and physical examination, cross-sectional imaging and measurement of serum markers, and the intervals were extended to six months after two years. We followed unresected patients in accordance with their clinical course.

All study sample characteristics were summarized with descriptive statistics. The primary endpoint of the study was to analyse the rate of resection. Secondary endpoints were the analysis of toxicity, overall survival (OS), progression-free survival (PFS), local control (LC) and metastases free-survival (MFS). The resection rate achieved in our previous prospective study of neoadjuvant chemoradiation was 33%^10^. Simon’s minimax two stage phase II design was planned to test the null hypothesis that the resection rate was ≤30% versus the alternative hypothesis that the resection rate was ≥50% with type I and II errors of 5% and 20% respectively. The study was designed to accrue a sample size of 39 patients with an interim analysis of 19 patients. Overall Survival was determined from the day of the histological diagnosis. Progression-free survival was obtained from the beginning of treatment to the observation of progression/recurrence, or to the last follow-up if no event was observed. OS and PFS curves were calculated with the Kaplan-Meier method.

This study was reviewed, approved and monitored by the independent Ethics Committee of our University. The methods were carried out in accordance with the approved guidelines. Signed informed consents were obtained from all study participants. This trial was registered at ClinicalTrials.gov with Identifier NCT02984501 on 2 December 2016.

## Results

### Patient Selection

From January 2012 through January 2015, forty-one patients (F:19; M:22) with histologically proven pancreatic ductal adenocarcinoma were accrued in the protocol. All patients showed a good performance status (ECOG Score 0) and none had undergone previous chemotherapy or radiotherapy.

All patients underwent a diagnostic laparoscopy to verify occult peritoneal and/or hepatic metastases and to perform peritoneal washing. Laparoscopy was positive (presence of hepatic and/or peritoneal metastases) in four patients (9.7%): two with peritoneal carcinomatosis, two with histologically proven hepatic metastases undetected on the CT scan. Moreover, all patients performed PET-CT with FDG resulting positive for distant disease in three patients (7.3%): two patients with pulmonary metastases, one patient with liver metastasis. According to the results of this pre-treatment workup, seven patients (17%) had metastatic disease and were therefore excluded from the protocol. Consequently, 34 patients were analysed.

Twenty-seven patients (80%) had locally advanced unresectable tumours. Of these, fifteen patients presented with encasement of the superior mesenteric artery, seven patients with encasement of the celiac axis and five patients with an occlusion of the portal vein that precluded vascular resection. Seven patients (20%) had borderline resectable disease, of which six patients on the basis of the involvementof less than 180 degrees of the superior mesenteric artery or involvement of the hepatic artery within 1 cm of the celiac axis and one patient on the basis of advanced regional adenopathy. The demographic and clinical characteristics of these patients are listed in Table 1.

### Treatment-Related Toxicity

Induction chemotherapy was well tolerated. The most common side effects during this phase were grade 1–2 haematological events; only one patient experienced grade 3 thrombocytopenia. Two patients (5.8%) developed grade 3 elevated transaminases. No grade 4 toxicity was observed. One patient developed peripheral sensory neuropathy. Table 2 details the profile of toxicity.

During the induction chemotherapy phase, the planned oxaliplatin and gemcitabine dose-intensities were 50 mg/mq/week and 500 mg/mq/week respectively. The administered dose-intensities of oxaliplatin and gemcitabine were 94% of the planned ones for both agents.

During radiochemotherapy, the most frequent all-grade toxicities were haematological (anemia (70.3%), leukopenia (74%), thrombocytopenia (70.3%), nausea (44.4%) and fatigue (37%). The most common events were hematologic. Grade 3 leukopenia and grade 3–4 thrombocytopenia occurred in seven patients (25.9%) and in nine patients (33.3%) respectively. There were no cases of grade 3–4 anemia. Gastrointestinal toxicity consisted of nausea (37% for grade 1 and 7.4% for grade 2), grade 1 vomiting (11.1%) and diarrhea (7.4% for grade 1, 3.7% for grade 2). One patient (3.7%) developed grade 3 of hypertransaminasaemia. Fatigue and anorexia of grade 1 occurred in 33.3% and 11.1% of patients respectively and grade 2 in 3.7% of patients. The toxicity data of RCT are summarized in Table 3. None of the hospitalizations during RCT were related to treatment. No toxic deaths were observed in this study. Overall, the treatment was well tolerated. Twenty-three patients (85%) completed RCT without any interruption. All patients with BRPC completed the combined treatment; four patients with LAPC interrupted treatment early at median total radiation dose of 51.3 Gy (at the doses of 55.8 Gy, 45 Gy, 57.6 Gy, 46.8 Gy, respectively) for the occurrence of cholangitis secondary to stent obstruction that required hospitalization and replacement of biliary stent. In the radiochemotherapy phase, the administered dose-intensity of gemcitabine was 78% of the planned dose-intensity of 600 mg/mq/week. Gemcitabine concurrent to radiotherapy was reduced or held because of toxicity in nine patients (33.3%).

### Treatment Efficacy

After two months of induction chemotherapy, all patients were evaluated for clinical response by using CT scan and PET-CT scan. Post-treatment CT scan showed that six patients (17.6%) had a partial response, in 23 patients (67.7%) the disease was stable and five patients (14.7%) experienced disease progression (two patients with local progression and liver metastases, three patients with local progression and peritoneal carcinomatosis). PET-CT scan showed a reduction of the maximum standard uptake value (SUVmax) in 12 patients (50%), in partial response and stable disease by CT scan, and also confirmed the progression of disease in five patients. Table 4 details the clinical response after induction chemotherapy both in borderline resectable and unresectable patients. Twenty-nine patients had no local and metastatic progression and remained in an acceptable general condition; two patients refused to continue the protocol; and twenty-seven patients received concomitant RCT.

All 27 patients were evaluated for clinical response. Post-treatment CT scan showed that seven patients (26%) had a further partial response, fifteen patients (55.5%) had stable disease and five patients (18.5%) experienced disease progression. If the post-treatment PET-CT scan is also taken into consideration, all responders patients showed a reduction of SUVmax. Four of the patients with stable disease at CT scan had a partial metabolic response, and five patients had a complete metabolic response at PET-CT scan. Table 5 shows the clinical response after RCT in the groups of borderline resectable and unresectable patients. Four patients (14.8%) with BRPC, with involvement of less than 180 degrees of the superior mesenteric artery, and 11 patients (40.7%) with LAPC, who presented with encasement of the superior mesenteric artery or the celiac axis, demonstrated a downstaging of their disease to resectable status after having received the combined treatment. Nineteen patients were evaluated with surgical exploration, and fifteen patients (55.5%) underwent surgical radical resection, with negative margins. No patient died due to perioperative complications. Seventeen patients (63%) were eligible to receive sequential gemcitabine-based chemotherapy for a median of six months (range, 3–12 months). In addition, nine patients (33.3%) with progressive disease received further cytotoxic chemotherapy after progression. The median follow-up for all patients was 20 months (range, 6.1 to 58.9 months). At the time of evaluation nine patients were alive with a median follow-up of 38.3 months, and five out of 27 patients were free from disease after surgery.

**Figure 1:**
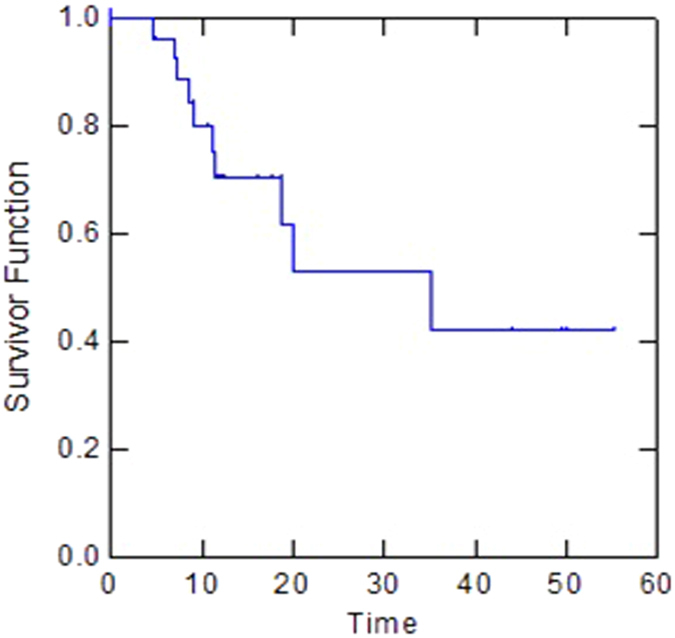
Kaplan-Meier method for Progression-Free Survival (PFS).

**Figure 2:**
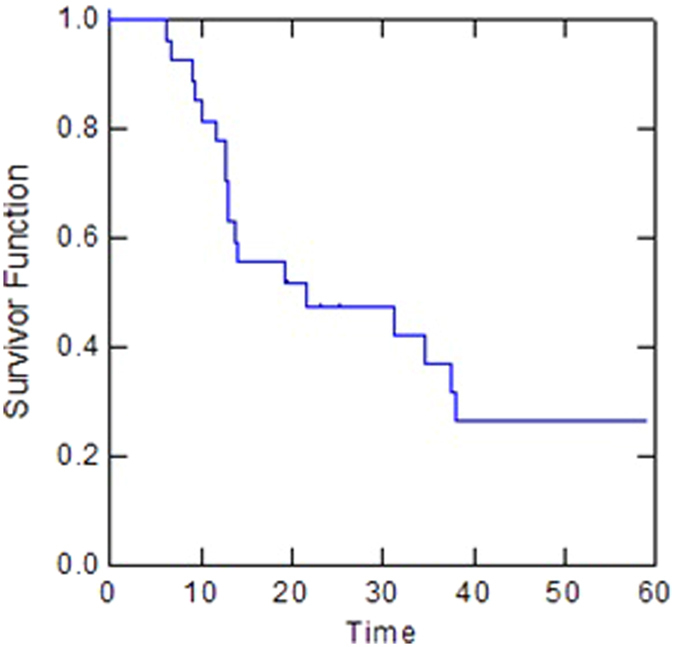
Kaplan-Meier method for Overall Survival (OS).

For the whole group, the median PFS was 20 months (95% CI: 0.25–0.74). One-year, two-year and three-year PFS were 70%, 53% and 42%, respectively (Fig. 1). The initial site of progression was at distant in eight patients and local and at distant in two patients. The median PFS for borderline resectable patients was 8.4 months compared with 35.2 months for unresectable patients (p < 0.01). Two-year and three-year metastases-free survival (MFS) was 53% and 42%, respectively (median, 20 months). Two-year and three-year local control (LC) were 95% and 79%, respectively. For the whole group, median OS was 19.2 months (95% CI: 0.31–0.68). One-year OS, two-year OS and three-year OS were 77%, 47% and 37%, respectively (Fig. 2). The median OS for borderline resectable patients was 21.5 months compared with 14 months for unresectable patients (p = 0.3). Patients who underwent resection had a significantly longer median OS compared with non resected patients (37.6 months vs 13 months, p = 0.03). The median PFS for resected patients was 35.2 months compared with 18.7 months for non resected patients (p = 0.3).

## Discussion

Approximately 30% of patients with pancreatic cancer have locally advanced disease defined as unresectable pancreatic cancer, without evidence of distant metastatic disease. This group of patients has been studied intensively in recent years, and neoadjuvant therapies have been proposed to achieve better local tumour control or tumour downstaging. In these patients, the conversion to resectability represents the ultimate goal of treatment, although actual downstaging of tumors that encase or obliterate celiac or superior mesenteric vessels is uncommon with current treatment strategies^8^. Over the past few years, due to technological progresses in the field of preoperative imaging, a distinct subset of patients in the locally advanced patient group has emerged: patients with BRPC. While this type of cancer is potentially resectable, patients with BRPC have a higher likelihood of incomplete resection. The use of neoadjuvant therapy in the setting of borderline resectable disease has been a highly debated topic. There is a growing body of literature to suggest that there is a potential role for neoadjuvant therapy to treat micrometastatic disease with chemotherapy, as well as for the treatment of local disease with radiotherapy. The evidence that R0 resection rates are higher after neoadjuvant therapies highlights the need for research in this specific field^9^. In our study, the enrolled patients had borderline resectable or unresectable disease.

In our opinion, the selection of patients affected by pancreatic cancer is a crucial issue in the debate of integrated treatments. As in one our previous study, in the diagnostic workup protocol we have combined imaging exams (CT scan, PET-CT scan) with laparoscopy in order to better select patients for RCT, as well as for their selection for surgery after the combined modality^10^. Seven patients (17%) were excluded from the protocol because of the evidence of metastatic disease at the pre-treatment staging; five patients experienced disease progression after induction chemotherapy and five patients after RCT.

The use of PET-CT with FDG for response evaluation after RCT in pancreatic carcinoma is not yet clinically established, however, there is increasing evidence that this imaging method is more reliable for overall response assessment^11^,^12^,^13^. Although PET is not anatomically accurate enough to predict vascular tumour contact, it may be a valid tool to select patients for explorative laparotomy in order to attempt surgical resection. In our study, all responder patients showed a reduction of SUVmax. Moreover, of those patients with stable disease at CT scan, four patients had a partial metabolic response and five patients had a complete metabolic response at PET-CT scan. Subsequently, nineteen patients were selected for surgical exploration.

In this phase II study, patients with BRPC and LAPC were treated with induction oxaliplatin and gemcitabine, followed by a high weekly dose of gemcitabine concurrent to radiation therapy. It is important to note that this treatment strategy was well tolerated. During the induction chemotherapy phase, the most common side effects were grade 1–2 haematological events. During radiochemotherapy, the most frequent all-grade toxicities were haematological, nausea and fatigue, which were controlled with appropriate medical therapy. The tolerability of RCT, after a short period of just two months of induction chemotherapy, is a critically important benefit of this strategy as it allows patients to complete the neoadjuvant therapy and maintain an appropriate weight and nutrition status prior to surgery. A well-tolerated neoadjuvant treatment approach may reduce surgical mortality and improve survival.

In the literature, retrospective studies have reported promising results from systemic induction chemotherapy followed by RCT in patients with LAPC who achieved disease control. A recent report published by the European Groupe Cooperateur Multidisciplinaire en Oncologie (GERCOR) revealed that patients with LAPC who did not progress after initial gemcitabine and went on to receive RCT had significantly longer OS than those who received chemotherapy alone (15 vs 11.7 months; p = 0.0009)^4^. The GERCOR study thus suggests there is a subgroup of patients who may benefit from the addition of RCT.

The GERCOR LAP 07 phase III study employed a 2 × 2 randomization to evaluate the benefit of chemoradiation after 4 months of induction gemcitabine, and also the benefit of adding erlotinib to the treatment regimen. With a median follow up of 36.7 months, the median overall survival was equivalent in the chemotherapy arm compared to the chemoradiation arm (16.5 versus 15.3 months, respectively; p = 0.8). Although in this study chemoradiotherapy did not increase overall survival compared with chemotherapy, increase in progression-free survival resulted in a long period without treatment (6.1 vs 3.7 months, p = 0.02) and less frequent locoregional tumor progressions (32% vs 46%, p = 0.04)^14^.

In the current study, we reported a median PFS of 20 months and a median OS of 19.2 months. One-year OS, 2-year OS and 3-year OS were 77%, 47% and 37%, respectively.

Recently, other investigators have evaluated the safety and efficacy benefit of oxaliplatin and gemcitabine-based induction chemotherapy followed by RCT in patients with LAPC. Crane *et al*. reported the results of a phase II trial of induction gemcitabine, oxaliplatin and cetuximab for two months followed by capecitabine and cetuximab-based RCT in 69 patients with LAPC (74% of patients had unresectable tumours, 26% had BRPC). Median overall survival time was 19.2 months and 1-year, 2-year, and 4-year actuarial overall survival rates were 66.0%, 25.02%, and 11.3%, respectively. Median PFS was 12.5 months^15^. Esnaola *et al*. evaluated in a phase II study the safety and efficacy of induction gemcitabine, oxaliplatin and cetuximab for six cycles followed by selective capecitabine-based RCT in 37 patients with BRPC or unresectable LAPC. With a median follow-up of 11.9 months for all patients, median PFS and OS were 10.4 months and 11.8 months respectively. In patients with unresectable tumours median OS was 9.3 months and in patients with BRPC median OS was 24.1 months^16^. Leone *et al*. reported the results of a trial of induction gemcitabine and oxaliplatin for two months followed by gemcitabine twice-weekly (50 mg/mq/daily), concurrent with radiotherapy in 15 patients with BRPC and 24 patients with unresectable disease. After a median follow-up of 13 months for the whole group, median PFS and OS were 10.2 months and 16.7 months respectively. OS was 27.8 months for patients with borderline resectable disease and 13.3 months for patients with unresectable disease (p = 0.045)^16^.

In a recent meta-analysis of phase II trials of neoadjuvant therapy for pancreatic adenocarcinoma, Assifi *et al*. found that of 134 patients with borderline and locally advanced tumours, 31.6% underwent resection following neoadjuvant treatment. Median survival was 22.3 months in resected patients, whereas OS in all patients was 11 months^9^. These findings are virtually similar to the observations in the above studies. While in the Esnaola *et al*.^16^ and in the Leone *et al*.^17^ studies, the resectability rates were 29.7% and 28% respectively, in our study 55.5% of patients underwent surgical resection after treatment protocol. Of the 15 patients who had curative resection, eleven were from the unresectable group, four from the borderline resectable status. All patients had radical resection (R0 resection). These results demonstrate a higher resection rate than shown in the other series and a more favorable R0 resection rate. Moreover, in the present study preoperative RCT did not increase perioperative morbidity or mortality, as has been previously reported elsewhere^18^.

In addition, we had excellent rates of local control in this series, with the majority of patients demonstrating stable disease in the high-dose radiation field in the unresected group of patients. Median overall survival was also longer than that contained in the historical data of the literature^19^,^20^, which is on average between 8 and 11 months, and similar to the results of the Crane *et al*. study^15^.

Unfortunately, distant metastatic disease continued to present a treatment challenge in these patients, with resultant detriment to progression-free survival in this series despite the high rates of local control, notwithstanding the median PFS was marginally extended compared with the literature. Overall, our data suggests that although loco-regional outcomes were excellent, systemic therapy still needs further optimization.

In conclusion, this study suggests that induction oxaliplatin and gemcitabine-based chemotherapy followed by concurrent RCT with a high weekly dose of gemcitabine is feasible, safe, and potentially facilitates resection of borderline resectable or locally advanced pancreatic cancers. The strengths of our study are the prospective design and the selection of patients. The study is limited by the small patient cohort. High rates of metastatic disease either before or after completion of definitive therapy suggests the need for further investigation into the biology of this disease and highlights the importance of optimizing systemic chemotherapy. We currently have an ongoing phase II trial at our institution exploring FOLFIRINOX chemotherapy followed by RCT in the locally advanced setting.

## Additional Information

**How to cite this article:** Fiore, M. *et al*. Phase II study of induction chemotherapy followed by chemoradiotherapy in patients with borderline resectable and unresectable locally advanced pancreatic cancer. *Sci. Rep.* **7**, 45845; doi: 10.1038/srep45845 (2017).

**Publisher's note:** Springer Nature remains neutral with regard to jurisdictional claims in published maps and institutional affiliations.

## Figures and Tables

**Table 1:** Demographic and clinical characteristics.

Characteristic	No of patients (N = 34)	%
Age (years)		
Median	63.5	
Range	40–75	
Sex		
Male	17	50
Female	17	50
ECOG performance status		
0	34	100
1	0	0
CA 19-9 at diagnosis, U/mL		
Median	998	
Range	<2–5750	
Tumor localization		
Head	29	85.3
Body/Tail	5	14.7
Resectability status		
Borderline resectable	7	20
Locally advanced unresectable	27	80

Abbreviation: ECOG, Eastern Cooperative Oncology Group.

**Table 2:** Toxicity profile of induction chemotherapy (N = 34 patients).

Toxicity	Grade 1		Grade 2		Grade 3		Grade 4		All Grades	
	No	%	No	%	No	%	No	%	No	%
Hematologic										
Anemia	10	29.4	4	11.7	0	0	0	0	14	41.1
Leukopenia	3	8.8	2	5.9	0	0	0	0	5	14.7
Thrombocytopenia	6	17.6	5	14.7	1	2.9	0	0	12	35.2
Gastrointestinal										
Nausea	8	23.5	1	2.9	0	0	0	0	9	26.4
Vomiting	1	2.9	0	0	0	0	0	0	1	2.9
Diarrhea	2	5.8	0	0	0	0	0	0	2	5.8
Liver and biliary										
Elevated total bilirubin	1	2.9	0	0	1	2.9	0	0	2	5.8
Elevated AST/ALT	6	17.6	3	8.8	2	5.8	0	0	11	32.2

**Table 3:** Toxicity profile of radiochemotherapy (N = 27 patients).

Toxicity	Grade 1		Grade 2		Grade 3		Grade 4		All Grades	
	No	%	No	%	No	%	No	%	No	%
Hematologic										
Anemia	11	40.7	8	29.6	0	0	0	0	19	70.3
Leukopenia	4	14.8	9	33.3	7	25.9	0	0	20	74
Thrombocytopenia	6	22.2	4	14.8	6	22.2	3	11.1	19	70.3
Gastrointestinal										
Nausea	10	37	2	7.4	0	0	0	0	12	44.4
Vomiting	3	11.1	0	0	0	0	0	0	3	11.1
Diarrhea	2	7.4	1	3.7	0	0	0	0	3	11.1
Liver and biliary										
Elevated total bilirubin	3	11.1	0	0	0	0	0	0	3	11.1
Elevated AST/ALT	2	7.4	2	7.4	1	3.7	0	0	5	18.5
Constitutional										
Fatigue	9	33.3	1	3.7	0	0	0	0	10	37
Anorexia	3	11.1	1	3.7	0	0	0	0	4	14.8

**Table 4:** Clinical response (by CT scan) after induction chemotherapy in borderline resectable and unresectable patients (N = 34 patients).

Response	Resectability Status	
	Borderline resectable group (7 pts)	Unresectable group (27 pts)
Partial Response	2	4
Stable Disease	4	19
Progression Disease	1	4

Pts: patients.

**Table 5:** Clinical response after radiochemotherapy in borderline resectable and unresectable patients (N = 27 patients).

Response	Resectability Status	
	Borderline resectable group (6 pts)	Unresectable group (21 pts)
Partial Response	3	4
Stable Disease	1	14
Progression Disease	2	3

Pts: patients.
